# ProPept-MT: A Multi-Task Learning Model for Peptide Feature Prediction

**DOI:** 10.3390/ijms25137237

**Published:** 2024-06-30

**Authors:** Guoqiang He, Qingzu He, Jinyan Cheng, Rongwen Yu, Jianwei Shuai, Yi Cao

**Affiliations:** 1 Postgraduate Training Base Alliance, Wenzhou Medical University, Wenzhou 325000, China; wuhewuhe1999@wmu.edu.cn; 2 Wenzhou Institute, University of Chinese Academy of Sciences, Wenzhou 325000, China; chengjy@wiucas.ac.cn (J.C.); rwyu@ucas.ac.cn (R.Y.); 3 Department of Physics, and Fujian Provincial Key Laboratory for Soft Functional Materials Research, Xiamen University, Xiamen 361005, China; qingzuhe@stu.xmu.edu.cn

**Keywords:** proteomics, retention time, ion intensity, ion mobility, multi-task learning, deep learning

## Abstract

In the realm of quantitative proteomics, data-independent acquisition (DIA) has emerged as a promising approach, offering enhanced reproducibility and quantitative accuracy compared to traditional data-dependent acquisition (DDA) methods. However, the analysis of DIA data is currently hindered by its reliance on project-specific spectral libraries derived from DDA analyses, which not only limits proteome coverage but also proves to be a time-intensive process. To overcome these challenges, we propose ProPept-MT, a novel deep learning-based multi-task prediction model designed to accurately forecast key features such as retention time (RT), ion intensity, and ion mobility (IM). Leveraging advanced techniques such as multi-head attention and BiLSTM for feature extraction, coupled with Nash-MTL for gradient coordination, ProPept-MT demonstrates superior prediction performance. Integrating ion mobility alongside RT, mass-to-charge ratio (*m*/*z*), and ion intensity forms 4D proteomics. Then, we outline a comprehensive workflow tailored for 4D DIA proteomics research, integrating the use of 4D in silico libraries predicted by ProPept-MT. Evaluation on a benchmark dataset showcases ProPept-MT’s exceptional predictive capabilities, with impressive results including a 99.9% Pearson correlation coefficient (PCC) for RT prediction, a median dot product (DP) of 96.0% for fragment ion intensity prediction, and a 99.3% PCC for IM prediction on the test set. Notably, ProPept-MT manifests efficacy in predicting both unmodified and phosphorylated peptides, underscoring its potential as a valuable tool for constructing high-quality 4D DIA in silico libraries.

## 1. Introduction

Mass spectrometry (MS) technology finds extensive application in proteomics research. The amalgamation of liquid chromatography (LC) and MS stands as a potent approach for the separation of complex compounds. However, the traditional proteomics methodologies that rely on DDA often encounter challenges stemming from the pace of MS sequencing and the semi-stochastic sampling of precursor ions, resulting in restricted throughput and diminished reproducibility [1]. DIA represents a progression and enhancement of the DDA mode, operating independently of prior MS scan outcomes to facilitate a relatively unbiased MS/MS analysis of all precursor ions within the defined isolation windows, thereby circumventing data imbalances stemming from randomness [2,3]. Nevertheless, a significant constraint remains in the current DIA proteomics approach, requiring the prior establishment of project-specific, high-quality spectral libraries through DDA analysis of extensively pre-fractionated or repetitively injected samples, leading to substantial time consumption and sample wastage [4,5].

In recent years, artificial intelligence has made considerable strides and found widespread application in the biomedical field, spanning medical image analysis [6,7,8], bioinformatics [9,10], disease diagnosis and treatment [11,12], and proteomics [13,14,15,16,17]. In proteomics research, we have reported several deep learning methods applicable to DIA data analysis, such as Dear-DIA^XMBD^ [13], an untargeted analysis method for directly analyzing DIA data; AttnPep [14], used for rescoring peptide-spectra match scores; and SeFilterDIA [15], designed to automatically identify high-confidence peptides. Deep learning approaches can also be leveraged to generate in silico libraries by predicting the fragment ion intensity, RT, and IM of given peptide sequences [18]. Noteworthy examples include DeepMass:Prism [19], Prosit [20], DeepDIA [21], pDeep [22], DeepPhospho [23], Guan et al. [24], MS2CNN [25], Predfull [26], and Deep4D [27]. Among these, DeepDIA employs a model based on bidirectional long short-term memory networks (BiLSTM) [28], encoding each amino acid into a 20-dimensional one-hot vector representing the 20 amino acids. Deep4D utilizes a deep learning model based on the self-attention [29] module, encoding each amino acid into a 23-dimensional one-hot vector, with an initial 20 dimensions representing the 20 amino acids and the subsequent three dimensions representing acetylation, oxidation, and phosphorylation modifications. Another example, DeepPhospho, also harnesses a model based on the self-attention module and exhibits outstanding performance in predicting phosphorylated peptide RT.

The combination of ion mobility spectrometry with mass spectrometry holds promise for enhancing sensitivity and simplifying spectra [30,31,32], particularly demonstrating unique advantages in the analysis of proteins, peptides, and complex compound isomers, thus propelling proteomics into a new era of 4D analysis. Calculating the inherent collisional cross-section (CCS) values of peptides based on experimentally derived ion mobility can significantly enhance the reliability of their identification [33]. Additionally, 4D DIA-based proteomics has shown higher precursor ion sampling rates and increased specificity in precursor identification [34]. Despite the notable progress achieved by deep learning methods in constructing in silico libraries, the construction of high-quality in silico libraries for 4D DIA proteomics and phosphoproteomics remains a challenge.

Here, we propose ProPept-MT, a new deep learning-based multi-task learning model, designed for the precise prediction of RT, ion intensity, and IM of both unmodified and phosphorylated peptides to construct 4D DIA in silico libraries. Through ProPept-MT, a workflow for 4D DIA proteomics analysis has been developed, based on the predicted multidimensional in silico libraries. ProPept-MT features a hybrid network architecture that merges self-attention modules and BiLSTM modules, bolstered by Nash-MTL [35] for gradient aggregation, thereby ensuring coordinated parameter updates across all tasks. Prominently, ProPept-MT indicates superior prediction performance with fewer training parameters compared to existing deep learning-based single-task prediction models.

## 2. Results

### 2.1. Development of Model Structure

ProPept-MT is a cutting-edge multi-task deep learning architecture designed to predict multiple peptide features simultaneously. We concatenated the multi-head attention module with the BiLSTM module to fully leverage their advantages in capturing global sequence information. The multi-head attention module serves as the core structure, establishing direct associations between different positions, and focusing on the interactions among individual amino acids within the sequence to enhance feature extraction capabilities. Its robust sequence modeling ability allows it to synchronously process information from various parts of the sequence, capture long-range dependencies, and improve computational efficiency through parallel processing across multiple attention heads, thereby reducing the likelihood of information loss. The BiLSTM, as an auxiliary structure, further augments the model’s performance by capturing longer-term temporal features. It learns from potential information overlooked by preceding subnetworks, generating rich and comprehensive context-aware representations through bidirectional sequence processing. This enhances the accuracy of predictions and robustness to different types of protein sequences.

The hybrid design demonstrates superior performance in deep learning methodologies. Compared to traditional approaches, this innovative combination can analyze the proteomics mass spectrometry data more comprehensively and in detail, which provides more reliable and precise data support for scientific research. Additionally, a series of single-task (ST) assessments were performed on a designated dataset using identical model specifications and hyperparameters, denoted as ProPept-ST, to enable comparative analysis with the multi-task model. The conclusive experimental findings manifest the exceptional performance of ProPept-MT over ProPept-ST.

### 2.2. Performance of ProPept-ST in Predicting Retention Time

Liquid chromatography is integral to mass spectrometry analysis in bottom-up proteomics [36], with RT playing a crucial role in DIA proteomics. To assess ProPept-MT’s performance, we initially validated ProPept-ST’s capacity for predicting RT. We compared ProPept-ST with existing single-task models for RT prediction, which can be applied to unmodified or phosphorylated peptides, thereby illustrating the sturdiness of its model architecture.

The study commenced with an examination of the predictive efficacy of RT for unmodified peptides, juxtaposing the performance of ProPept-ST against three contemporary deep learning models: Deep4D, DeepDIA, and DeepLC. DeepLC incorporates a deep convolutional neural network (CNN) architecture with an encoding approach grounded in atom composition. The evaluation hinged on 15 unmodified peptide datasets as delineated in the DeepLC study, where peptides were characterized by experimental RT or indexed RT (iRT) [37]. These datasets spanned three distinct LC modalities: reversed-phase LC (RPLC), hydrophilic interaction LC (HILIC), and strong cation exchange chromatography (SCX). ProPept-ST underwent preliminary pre-training on the SWATH library dataset, followed by fine-tuning on the remaining 14 datasets.

**Figure 1:**
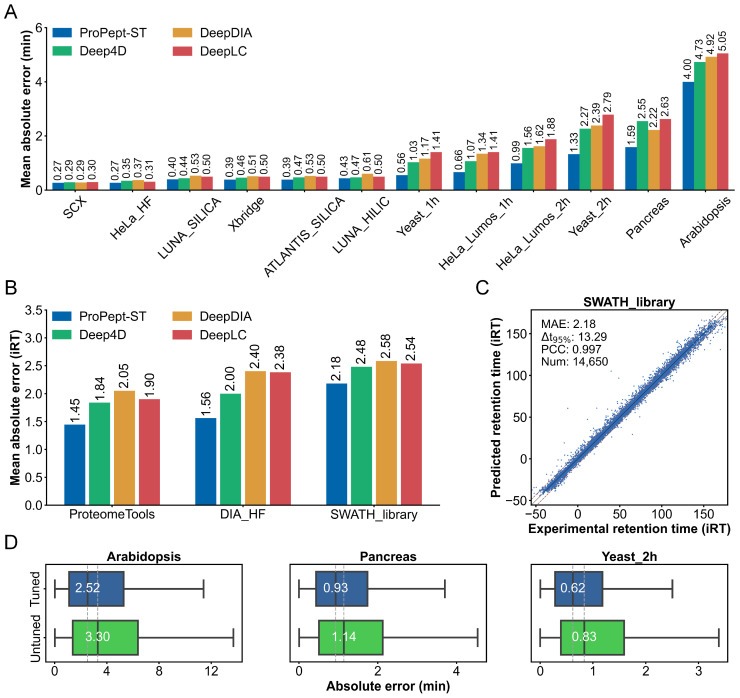
ProPept-ST evaluates the prediction performance of unmodified peptide retention time. (**A**,**B**) The prediction accuracy of different models for peptide RT (**A**) and iRT (**B**) is compared based on the mean absolute error on various datasets. (**C**) Scatter plot comparing ProPept-ST predicted RT values with experimentally observed RT values for the SWATH library dataset. (**D**) Distribution of absolute errors in peptide RT prediction by the ProPept-ST model, both fine-tuned and retrained.

Across all 15 datasets, ProPept-ST consistently outperformed extant models in terms of mean absolute error (MAE) (Figure 1A,B) and $\Delta t_{95\%}$ (Supplementary Figure S1). Importantly, the HeLa HF dataset, constructed using 15-min short gradients, posed a potential challenge due to reduced resolution and peak capacity, potentially affecting the predictive accuracy of apex peptide RT [38]. Despite these challenges, ProPept-ST exhibited superior performance compared to the leading DeepLC model on the HeLa HF dataset, achieving MAE values of 0.27 vs. 0.31 and $\Delta t_{95\%}$ values of 1.46 vs. 1.62. In the case of the SWATH library test set, the predicted iRT values demonstrated high precision, with a PCC of 0.997 (Figure 1C).

Furthermore, we subjected ProPept-ST to retraining on the 14 datasets excluding the SWATH library. Figure 1D and Figure S2 indicate that, among the 14 test datasets, the proportion of cases where the fine-tuned median absolute error (MedAE) exceeds that of the non-fine-tuned results is 71.4%, with only 0.07% of cases showing a decrease in performance. This highlights the utility of fine-tuning in enhancing model adaptability to diverse LC types and gradients, thereby underscoring its considerable benefits in RT prediction. Collectively, these findings showcase the superior predictive performance of ProPept-ST over other established models for unmodified peptide RT prediction.

**Figure 2:**
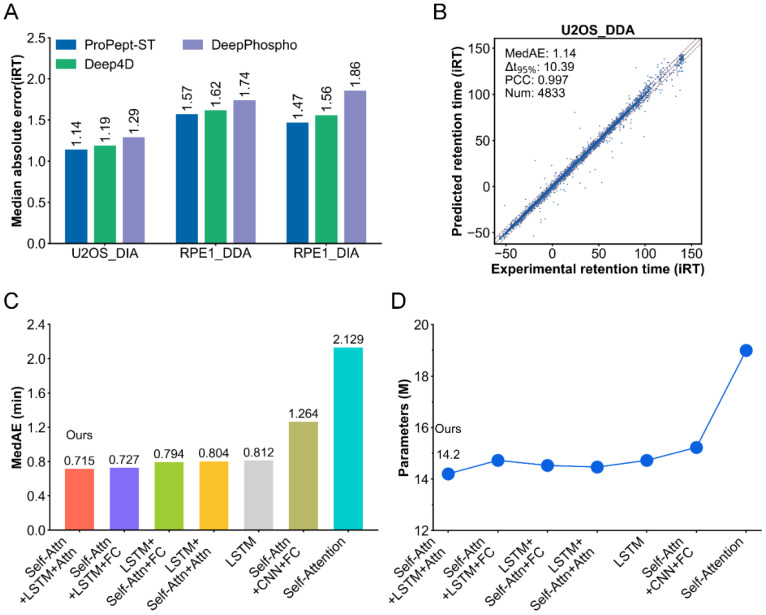
ProPept-ST assesses the performance of predicting phosphorylated peptide RT and the ablation experiment on the ProPept-ST model. (**A**) Comparison of the prediction accuracy of various models for phosphorylated peptide RT based on the median absolute error on different datasets. (**B**) Scatter plot comparing ProPept-ST-predicted iRT values with experimentally observed iRT values for the U2OS_DDA dataset. (**C**) Median absolute error of RT prediction by ProPept-ST and six other models on the benchmark dataset H4 DDAp. (**D**) Parameter count comparison between ProPept-ST and six other models.

To evaluate ProPept-ST’s performance in predicting RT for phosphopeptides, we conducted a comparative analysis against DeepPhospho and Deep4D, utilizing three distinct phosphopeptide RT datasets: RPE1 DDA, RPE1 DIA, and U2OS DDA, following the methodology described in the DeepPhospho study. ProPept-ST undergoes pre-training on the RPE1 DDA dataset, followed by fine-tuning on the RPE1 DIA and U2OS DIA datasets. ProPept-ST exhibited superior performance in terms of MedAE across all three datasets, as depicted in Figure 2A. Specifically, on the RPE1 DDA test set, ProPept-ST achieved a MedAE of 1.57, surpassing Deep4D and DeepPhospho, which scored 1.62 and 1.74, respectively. Moreover, on the U2OS_DDA test set, the predicted iRT values closely matched the experimentally observed iRT values, presenting high precision with a PCC of 0.997 (Figure 2B). These findings highlight ProPept-ST’s remarkable capability in accurately predicting phosphopeptide RT.

### 2.3. Ablation Studies

To elucidate the excellent design of our model, we executed a comparative analysis contrasting ProPept-ST with six alternative models (Figure 2C,D). These models predominantly feature combinations of self-attention modules, LSTM networks, and CNN modules. We meticulously designed and tested various parameter combinations, with Table 1 showcasing the main combinations. Each module’s layer count was carefully adjusted to ensure optimal performance. In the final stages of the model, we integrated either attention modules or multi-layer perceptron (MLP) layers to further enhance performance. Through this detailed tuning and optimization, we aimed to achieve the highest possible effectiveness in all aspects. Performance evaluation was carried out on the H4 DDAp’s RT dataset, with reported MedAE values. The finding reveals that in our model architecture, integrating attention modules at the end supersedes the direct use of fully connected layers, yielding MedAE values of 0.715 and 0.727 (Figure 2C), respectively. Interestingly, despite possessing the largest parameter count among these models, the model utilizing solely the self-attention modules exhibited the poorest performance. Noticeably, ProPept-ST showed outstanding performance with the fewest parameters (Figure 2D).

### 2.4. Performance of ProPept-MT on Benchmark Datasets

Based on our research, it is argued that fine-tuning a pre-trained model generally yields superior performance compared to retraining from scratch. As such, we initially pre-trained on the H5 DDAp dataset and subsequently fine-tuned on eight other specific datasets. Simultaneously, ProPept-ST trained individually for each task on the benchmark datasets is regarded as the evaluation baseline.

**Figure 3:**
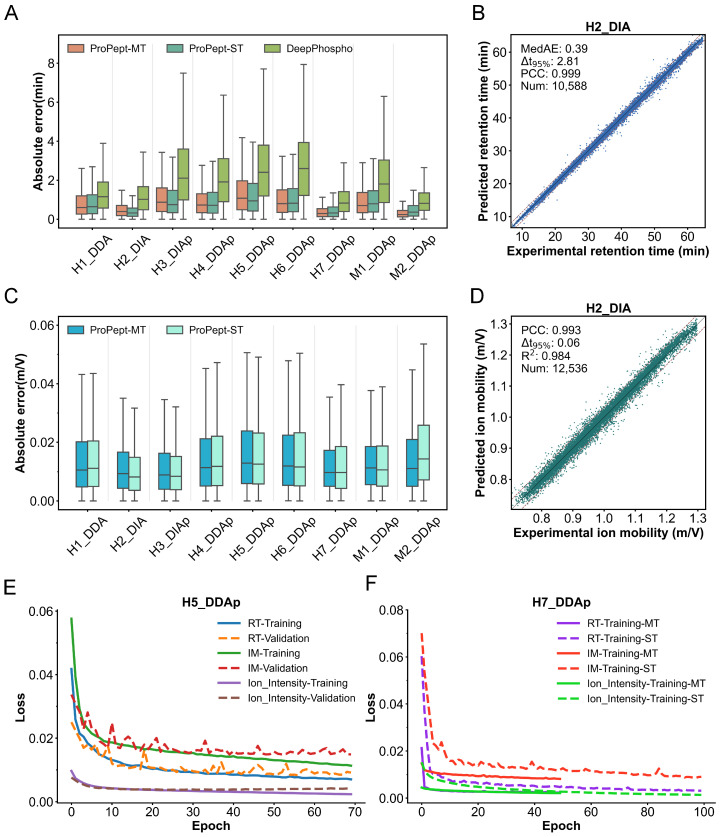
ProPept-MT’s performance in predicting RT and IM, as well as the loss curves for training three tasks on specific datasets. (**A**) Distribution of absolute errors for predicting RT on benchmark datasets for each model. (**B**,**D**) Scatter plots showing ProPept-MT’s predictions of RT (**B**) and IM (**D**) on the H2 DIA test set. (**C**) Distribution of absolute errors for predicting IM on benchmark datasets for ProPept-MT and ProPept-ST. (**E**) Loss curves for training and validation of the three tasks on dataset H5 DDAp for ProPept-MT. (**F**) On the H7 DDAp training set, the loss curves of ProPept-ST retrained on three tasks and the fine-tuned loss curves of ProPept-MT.

For the RT prediction task, ProPept-MT surpasses previously reported models on five datasets in terms of MedAE (Figure 3A). Specifically, on the H1 DDA test set, ProPept-MT achieves a MedAE of 0.598, surpassing the performance of both ProPept-ST and DeepPhospho, which achieve MedAEs of 0.643 and 1.157, respectively. In all benchmark datasets, all values are presented in minutes within the original RT dimension. Additionally, the PCC value on the H2 DIA test set is exceptionally high, reaching 0.999 (Figure 3B and Figure S3). Furthermore, in accordance with the primary indicator highlighted in the DeepPhospho study for RT prediction, we compared $\Delta t_{95\%}$ values (Supplementary Figure S4A). ProPept-MT outperforms both ProPept-ST and DeepPhospho on nine datasets, demonstrating performance on the H1 DDA test set of 4.97 compared to 5.20 and 6.59, respectively. Strikingly, for DeepPhospho, five Transformer encoder layers (4–8) of varying sizes were trained and integrated for testing.

For the IM prediction task, Table 2 showcases that across five datasets, ProPept-MT’s PCC values exceed those of ProPept-ST, achieving a performance of 0.992 compared to 0.981 on the M1 DDAp test set. Moreover, Figure 3C portrays the distribution of IM absolute errors between ProPept-MT and ProPept-ST. The high accuracy of IM prediction, with a PCC value of 0.993, is further revealed on the H2 DIA test set (Figure 3D and Figure S5).

**Figure 4:**
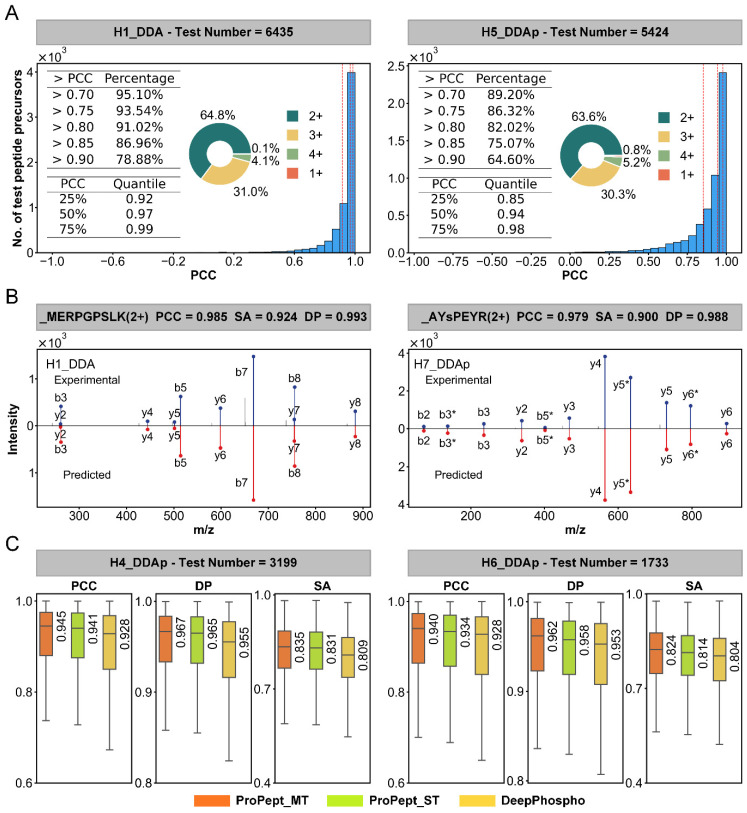
Performance of ProPept-MT in predicting fragment ion intensity. (**A**) Histogram distribution of PCC for each peptide on the H1 DDA and H5 DDAp test sets. (**B**) Mirror plot showing the experimental and predicted values of fragment ion intensities for two specific peptides (unmodified peptide and phosphopeptide). (**C**) Box plots showing the distribution of PCC, DP, and SA for ProPept-MT on the H1 DDA and H6 DDAp test sets.

For the fragment ion intensity prediction task, ProPept-MT outperforms ProPept-ST and DeepPhospho on nine datasets in terms of median PCC, as shown in Table 2, achieving a performance of 0.941 vs. 0.927 vs. 0.918 on the M2 DDAp test set. Of note is ProPept-MT’s performance on the H1 DDA and H5 DDAp test sets, where it achieves PCC values exceeding 0.75 for 93.54% and 86.32% of peptides, respectively, with median PCC values of 0.97 and 0.94 (Figure 4A). Mirror representations for specific peptides evidenced robust concordance between our prognostication and authentic measurement, with PCC values of 0.985 and 0.979, respectively (Figure 4B). In comparison to existing models, our multi-task model also exhibits improved overall consistency between experimental and predicted fragment ion intensities for the test set (Figure 4C). For the H4 DDAp dataset, ProPept-MT achieves a median PCC of 0.945, median spectral angle (SA) of 0.835, and median dot product (DP) of 0.967. Similarly, for the H6 DDAp dataset, ProPept-MT achieves a median PCC of 0.940, median SA of 0.824, and median DP of 0.962. Additionally, ProPept-MT outperforms DeepPhospho with respect to median SA on six datasets (Supplementary Figure S4B), which serves as the primary indicator for fragment ion intensity prediction in the DeepPhospho study.

Figure 3E presents the loss of ProPept-MT on both the training and validation sets of the H5 DDAp dataset, elucidating the model’s robust training and lack of overfitting. In Figure 3F, we display the fine-tuning loss of ProPept-MT on the H7 DDAp training set, comparing it with the loss of the ProPept-ST model trained separately for each of the three tasks. These results indicate that the loss for each task can be rapidly minimized by ProPept-MT during a brief training period. Detailed performance metrics are available in Table 2. Our findings disclose that, compared to the reported models, ProPept-MT can achieve optimal performance with the fewest parameters, underscoring its superior ability to predict peptide features.

### 2.5. Performance Comparison between ProPept-MT and Other Models

To assess the accuracy and fairness of the ProPept-MT model, the identical datasets employed for training, validation, and testing in the comparative models are utilized. Two deep learning models, DeepDIA and DeepPhospho, which are proficient in predicting features of unmodified peptides and phosphorylated peptides, will be compared. Although both models employ similar or slightly enhanced network structures for predicting two or more peptide features, their training methodologies involve training each task separately.

The data processing approach of comparative models is being followed, with the corresponding datasets being obtained for each model and the results being reported according to the evaluation metrics of the respective tasks. For instance, the capability of DeepDIA to predict RT and fragment ion intensity will be utilized. Peptides with lengths less than seven or greater than fifty, or those containing variable modifications, are filtered out. For the task of predicting fragment ion intensity, the selection is further refined to include only sub-ions with intensity values greater than zero and charge states of 1+ or 2+. Additionally, peptides where the number of sub-ions contained in each parent ion is greater than or equal to six are selected. Subsequently, peptides with precursor charges of 2+ and 3+ are segregated for DeepDIA training. In contrast, they will be treated as two separate tasks for joint training by ProPept-MT. One-third of the dataset is allocated for testing, while the remaining two-thirds are further divided into two-thirds for training and one-third for validation.

Similarly, DeepPhospho, like DeepDIA, can predict RT and fragment ion intensity but extends its support to phosphopeptides. The dataset used by DeepPhospho mirrors benchmark datasets, divided into training, validation, and testing sets in an 8:1:1 ratio. Consequently, the comparison results between ProPept-MT and DeepPhospho can be found in Section 2.4. Notably, in comparison with DeepDIA, we continue to fine-tune the filtered dataset using the parameters pre-trained by ProPept-MT on the benchmark dataset H5 DDAp. Conversely, DeepPhospho and DeepDIA undergo retraining on each dataset using their default parameters.

For the fragment ion intensity prediction task, a nuanced comparison with the DeepDIA model reveals 12 distinctive combinations of fragment ion types. These combinations are characterized by varying factors, including the charge states of sub-ions (1+ or 2+), the presence or absence of b/y ions, and the potential for two neutral losses (NH3 or H2O). Similarly, for the DeepPhospho model, eight unique combinations of fragment ion types are unveiled by our analysis. These combinations stem from key factors such as the charge states of sub-ions (1+ or 2+), the presence or absence of b/y ions, and the potential for the loss of H3PO4. Furthermore, our methodology involves the deliberate exclusion of loss items from the phosphate salt component that are deemed implausible. This rigorous approach includes a filtering mechanism to exclude the intensity predictions of these ions, ensuring the robustness and accuracy of our findings.

**Figure 5:**
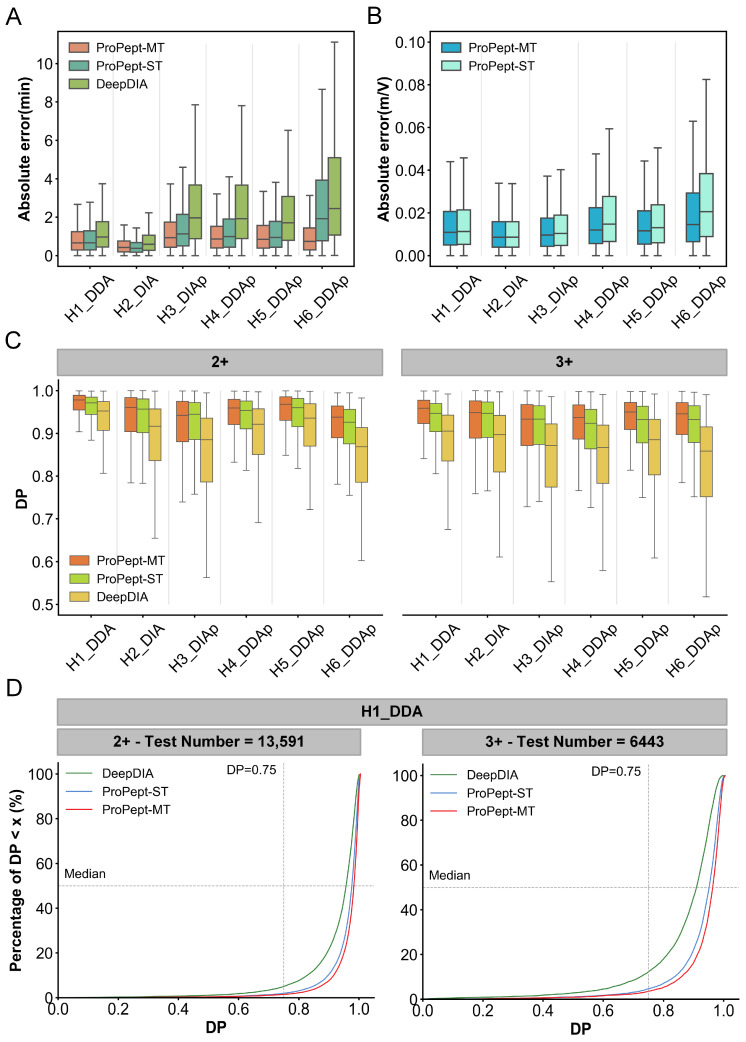
Comparing the performance of ProPept-MT and DeepDIA in predicting RT, IM, and fragment ion intensity. (**A**) Distribution of absolute errors for predicting RT on different datasets for each model. (**B**) Distribution of absolute errors for predicting IM on different datasets for ProPept-MT and ProPept-ST. (**C**) Assessing ProPept-MT’s performance in predicting fragment ion intensity for different precursor charges on benchmark datasets. (**D**) Distribution of dot product (DP) for predicting fragment ion intensity of 2+ and 3+ precursor charges on the H1 DDA test set for each model.

To ensure consistent comparisons, datasets were initially filtered to exclude those with insufficient data volume, which could potentially affect the performance of single-task training in DeepDIA. For result evaluation, the primary metrics outlined in the DeepDIA study are followed, with PCC and median DP being utilized as key evaluation metrics for RT and ion intensity, respectively. Concerning RT prediction, ProPept-MT outperforms both ProPept-ST and DeepDIA in PCC on five datasets, achieving 0.987 compared to 0.971 and 0.958 on the H6 DDA test set (Table 3). Figure 5A visualizes the distribution of absolute errors for RT prediction across six datasets, showing that ProPept-MT’s MedAE consistently exceeds that of DeepDIA and ProPept-ST on five datasets.

Furthermore, ProPept-MT showcases exceptional predictive performance in IM prediction, exhibiting a superior PCC compared to ProPept-ST across five datasets, achieving 0.971 versus 0.963 on the H6 DDAp test set (Table 3). In addition, ProPept-MT consistently outperforms various alternative baselines in IM prediction (Figure 5B). Moreover, in terms of median DP, ProPept-MT consistently surpasses the models reported in previous studies (Figure 5C). During fine-tuning, ProPept-MT takes advantage of fragment ions with H3PO4 loss from the pre-training set and fragment ions with two types of neutral losses (NH3 or H2O) in the fine-tuning set, achieving better performance than single-task approaches and highlighting the model’s generalization capability. Figure 5D portrays the distribution of DP values for each peptide in the test sets of the H1 DDA dataset, stratified by precursor charges of 2+ and 3+. For peptides with a precursor charge of 2+, the percentage surpassing a DP value of 0.75 is 98.53%, with a median DP of 0.978. Subsequently, for peptides with a precursor charge of 3+, the percentage is 96.35%, with a median DP of 0.959. For a detailed analysis of performance metrics, please refer to Table 3, where ProPept-MT’s performance across six datasets exceeds that of the reported models, underscoring its superior capability in predicting unmodified peptide features.

## 3. Materials and Methods

### 3.1. Dataset Collection and Pre-Processing

Constructing an effective benchmark dataset stands as a critical endeavor for training and assessing deep learning models. Curating multiple recently released raw mass spectrometry datasets from esteemed repositories, including ProteomeXchange [39], PRIDE [40], iProX [41], and jPOST [42], was initially undertaken. These MS data, acquired using timsTOF Pro or timsTOF Pro 2 mass spectrometers, provided crucial ion mobility information. Following that, two software packages, MaxQuant (version 2.4.8.0) [43] and DIA-NN (version 1.8.1) [44], were deployed to analyze MS data, thereby resulting in the final peptide identification outcomes. This meticulous process led to the compilation and assembly of nine benchmark datasets, comprising a total of 353,052 entries (Table 4). These datasets encompass samples from both humans and mice, featuring a range of variable modifications, such as phosphorylation at serine, threonine, and tyrosine sites, oxidation of methionine, and N-terminal acetyl modification. Moreover, carbamidomethylation of cysteine served as a fixed modification.

**Table 4:** Dataset Structure.

Data Name	Species	Instrument	Peptides	Identifier
H1_DDA	Human	timsTOF Pro	64,358	PXD041421 [45]
H2_DIA	Human	timsTOF Pro	125,360	PXD041391 [45]
H3_DIAp	Human	timsTOF Pro	42,351	PXD034709 [46]
H4_DDAp	Human	timsTOF Pro	31,599	PXD034709
H5_DDAp	Human	timsTOF Pro	42,677	PXD027834 [47]
H6_DDAp	Human	timsTOF Pro	16,784	PXD042842 [48]
H7_DDAp	Human	timsTOF Pro 2	9495	PXD043026 [49]
M1_DDAp	Mouse	timsTOF Pro	12,132	PXD028051 [50]
M2_DDAp	Mouse	timsTOF Pro 2	8296	PXD043026

Benchmark datasets exclusively comprise the 20 common amino acids. In the identification results of MaxQuant and DIA-NN, peptide data with *q* values exceeding 0.01 were filtered out. For the output files of MaxQuant, msms.txt and evidence.txt were specifically selected, and peptides with phosphorylation modification site probabilities below 0.75 were excluded to ensure dataset quality. Furthermore, given that these data originate from multiple Liquid Chromatography-Tandem Mass Spectrometry (LC-MS/MS) runs, the highest-scoring data point from the same peptide was selected for the fragment ion intensity prediction task, while fragment ions with intensities less than or equal to zero were excluded. The median of the corresponding target values was utilized for the RT and IM prediction tasks.

### 3.2. The Model Architecture of ProPept-MT

**Figure 6:**
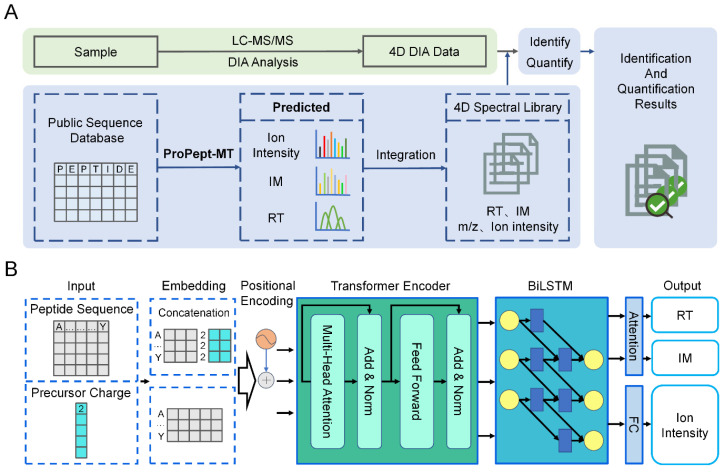
The workflow and model architecture of ProPept-MT. (**A**) ProPept-MT employs a multi-task deep learning model to generate in silico prediction libraries from protein or peptide sequence databases. (**B**) ProPept-MT is used for predicting RT, IM, and fragment ion intensity for any given unmodified peptide or phosphopeptide. Given the peptide sequence and precursor charge as input, our model uses Transformer encoder modules and a BiLSTM network to calculate context representations for all amino acids, which it finally outputs through separately designed output layers for each task.

Figure 6A depicts the workflow of ProPept-MT. ProPept-MT is a blended network structure that adopts multi-task deep learning to map peptide sequences into high-dimensional vectors. This intricate process involves self-attention modules, nonlinear transformations, and BiLSTM networks integrated into four main modules: an input layer, an embedding layer, a sequence modeling layer, and an output layer (Figure 6B). The embedding layer encodes both the input amino acid sequence and the precursor charge into feature vectors. Subsequently, the sequence modeling layer learns representations of peptide features, culminating in the generation of prediction values by the output layer. This integration enables the model to focus on inter-amino-acid correlations, capture long-term temporal features and latent information, and generate richer contextual information representations.

Each submodule is described as follows:

**Input layer.** A peptide consists of an amino acid sequence, with the precursor charge represented as a scalar. The 20 common amino acids are denoted in uppercase letters, such as “G” for glycine and “A” for alanine. If the N-terminal of the peptide contains an acetylation modification, “a” is prepended to the sequence; otherwise, “_” is prepended. Variable modifications in the sequence are indicated by “s,” “t,” and “y” for phosphorylation modifications at serine, threonine, and tyrosine sites, respectively, and “m” for methionine oxidation. “$” is appended at the end of the sequence to aid the model in determining when to cease processing the sequence. The maximum peptide length is set to 52, with any portion of the sequence shorter than 52 padded with “#”.

**Embedding layer.** For the RT prediction task, each amino acid is directly embedded into a 256-dimensional tensor. Conversely, for the IM and fragment ion intensity prediction tasks, each amino acid is first embedded into a 192-dimensional tensor and each precursor charge into a 64-dimensional tensor, which are then concatenated into a 256-dimensional tensor. To incorporate the positional information of amino acids, standard sine and cosine functions are used as positional encoding [29], resulting in a 54 × 256 tensor.

**Sequence modeling layer.** Serving as the backbone of ProPept-MT, this layer comprises a series of Transformer encoders and BiLSTM subnetworks. The Transformer encoder subnetwork enlists more efficient self-attention modules to capture correlations between amino acids at different positions in the peptide sequence. It consists of six stacked encoder layers, each containing a multi-head attention module and a fully connected feed-forward network, with residual connections and layer normalization. The number of attention heads and hidden layer dimensions of the feed-forward network are eight and 1024, respectively. The goal of this subnetwork is to extract the initial representation of the peptide and feed it to the next subnetwork. The BiLSTM subnetwork consists of a single bidirectional LSTM layer with 512 hidden dimensions and its goal is to capture longer distance dependencies more effectively. Furthermore, as a shared layer, the sequence modeling layer shares its learning parameters across tasks. The attention module is described as:

(1) $$Attention\left(Q,K,V\right)=Softmax\left(\frac{QK^{T}}{\sqrt{d_{k}}}\right)\cdot V$$

where Q,K,V is derived from the dot product between the input matrix and three parameter matrices. The operation $QK^{T}$ generates a similarity matrix between each amino acid’s position and other amino acids’ positions. Subsequently, each element in the matrix is divided by a scalar $\sqrt{d_{k}}$, followed by the application of the softmax function to generate probabilities. Finally, the result is multiplied by V to obtain the context vector representation for each amino acid. $d_{k}$ represents the size of the hidden layer.

**Output layer.** A linear layer is used to project the features of each amino acid position into an n-dimensional vector, which acts as the output for predicting fragment ion intensities. Here, n represents the number of fragment ion types to be predicted. For the RT and IM prediction tasks, the hidden layer outputs of BiLSTM are used for generating instance-specific weights for sequence features, and a weighted averaging approach is exercised to produce the final RT and IM predictions.

### 3.3. Loss Function

Two distinct loss functions were exploited to minimize training errors, the mean squared error (MSE) loss function for predicting fragment ion intensity and the L1 norm for predicting RT and IM. These functions are expressed as follows:

(2) $$\mathrm{MSE}=\frac{1}{n}\sum_{i=1}^{n}{\left(y_{i}-\hat{y_{i}}\right)}^{2}$$

(3) $$L1\;\mathrm{Loss}=\frac{1}{n}\sum_{i=1}^{n}\left|y_{i}-\hat{y_{i}}\right|$$

where n represents the number of training samples, $y_{i}$ is the experimental value, and $\hat{y_{i}}$ is the predicted value.

### 3.4. Model Training

The experiment was conducted using Python 3.9 and implemented within the Torch deep learning framework (version 1.10.0) [51] (https://pytorch.org/). Applying multi-task learning during model training offers a potential avenue for reducing computational costs, albeit accompanied by the challenge of potential conflicts arising in the gradients of distinct tasks. To tackle this issue, Nash-MTL, as delineated in Algorithm 1, approaches the gradient aggregation step as a bargaining game [35]. This methodology facilitates task negotiation, aiming to achieve consensus on the direction of updating shared parameters, thus effectively alleviating this challenge.

**Algorithm 1.** Nash-MTL**Input:** $\theta_{0}$- initial parameter vector, ${\left\{l_{i}\right\}}_{i=1}^{K}$–differentiable loss functions, μ–learning rate**Output:** $\theta^{T}$**for** *t* = 1,…, *T* **do** Compute task gradients $g_{i}^{t}=\nabla_{\theta (t-1)}l_{i}$ Set $G^{\left(t\right)}$ the matrix with columns $g_{i}^{\left(t\right)}$ Solve for $\alpha :\;{\left(G^{t}\right)}^{T}(G^{t})\alpha =1/\alpha$ to obtain $\alpha^{t}$ Update the parameters $\theta^{\left(t\right)}=\theta^{\left(t\right)}-\mu *G^{\left(t\right)}\alpha^{\left(t\right)}$
**end for**
**return** $\theta^{T}$

For the multi-task learning paradigm involving parameters θ, this methodology postulates the existence of a sphere $B_{\in }$ centered at the origin with a radius ∈. The objective is to locate the update vector Δθ within this defined sphere. This scenario is framed as a bargaining problem, where the center of the sphere represents the point of disagreement, while $B_{\in }$ signifies the set of agreements. The utility function for each participant is defined as $u_{i}(\Delta \theta )=g_{i}^{T}\Delta \theta$, where $g_{i}$ signifies the gradient vector of task i loss at θ.

A unified training approach was implemented, wherein each training iteration sequentially addresses distinct tasks: fragment ion intensity, RT, and IM. For the fragment ion intensity prediction task, should the need arise to train peptides of varying precursor charges separately, the sequence follows 3+, 2+, RT, and IM. The Adam gradient descent algorithm was applied uniformly across all tasks, characterized by a batch size of 128, beta1 of 0.9, beta2 of 0.999, epsilon of 1e-8, and a learning rate of 1e-4. Moreover, extensive exploration of hyperparameters was conducted, accompanied by model simplification. This endeavor facilitated ProPept-MT in capturing intricate features among amino acid sequences, thereby enhancing prediction precision.

### 3.5. Evaluation Metrics

For the fragment ion intensity prediction task, the median PCC was selected as the ultimate evaluation metric. In addition, to promote comparisons with other established models, we adhered to their evaluation criteria, incorporating normalized SA and DP as two supplementary metrics, and reported their respective medians. The definition of SA is as follows:

(4) $$SA=1-\frac{2*\arccos(y\cdot \hat{y})}{\Pi }$$

where $\hat{y}$ and y are the predicted and experimental vectors, respectively, with L2 norm equal to 1.

For the RT prediction task, the MedAE served as the primary evaluation metric, complemented by the coefficient of determination ($R^{2}$), inter quartile range (IQR), PCC, and $\Delta t_{95\%}$ for comparative analysis across models. Here, $\Delta t_{95\%}$ denotes the minimum time window accommodating 95% of peptides, reflecting the disparity between experimentally observed and predicted RT. Regarding IM prediction, we focused on $R^{2}$, PCC, and $\Delta t_{95\%}$ as key metrics, with PCC assuming the role of the principal evaluation criterion. The definitions of MedAE and $\Delta t_{95\%}$ are outlined as follows:

(5) $$MedAE=median(|y_{1}-\hat{y_{1}}|,\ldots ,|y_{i}-\hat{y_{i}}|)$$

(6) $$\Delta t_{95\%}=2*|y-\hat{y}{|}_{95\%}$$

where n represents the number of training samples, $y_{i}$ is the actual value, and $\hat{y_{i}}$ is the predicted value. The subscript 95% indicates that the deviation covers 95%.

In the context of ProPept-MT training, where multiple evaluation metrics are relevant to each task, a specific formula is used to gauge model performance, quantifying the extent of performance enhancement between successive training epochs. The formula is as follows:

(7) $$\Delta_{p}=100\%\times \frac{1}{T}\sum_{t=1}^{T}\frac{1}{M_{t}}\sum_{m=1}^{M_{t}}\frac{{\left(-1\right)}^{w_{t,m}}(B_{t,m}-N_{t,m})}{N_{t,m}}$$

where T represents the number of tasks; W represents the number of metrics; $w_{t,m}$ represents the optimization direction of the m metric of the t task, with a binary value where 0 indicates that the smaller the metric, the better, and 1 indicates that the larger the metric, the better; B represents the list of metrics for the first training epoch; N represents the list of metrics for the current training epoch.

## 4. Discussion

In this study, we introduced ProPept-MT, a new multi-task deep learning model designed to enhance the accurate prediction of peptide features and expedite 4D DIA proteomics by precisely predicting the RT, fragment ion intensity, and IM of unmodified peptides or phosphopeptides. First, the evaluation of ProPept-ST’s RT prediction performance was conducted using 15 unmodified RT datasets and three phosphopeptide RT datasets, comparing its performance with the existing advanced RT prediction models. Subsequently, the multi-task prediction performance of ProPept-MT on nine benchmark datasets was assessed and compared with ProPept-ST and the existing advanced models. Evidently, ProPept-MT demonstrates superior prediction performance across all datasets and can predict peptide features such as ion mobility, which other models cannot predict.

In evaluating ProPept-ST, we assessed the ability of single-task models to predict retention time for both unmodified and phosphorylated peptide data. ProPept-ST was compared with Deep4D, DeepDIA, and DeepLC for unmodified peptides, and with Deep4D and DeepPhospho for phosphorylated peptides. To ensure fairness, we used datasets from the respective studies: unmodified peptide data from DeepLC and phosphorylated peptide data from DeepPhospho. This approach was necessary due to the varying nature of the input data supported by these models. Notably, Deep4D, DeepPhospho, and DeepLC support modifications such as methionine oxidation, phosphorylation at STY sites, and N-terminal acetylation, whereas DeepDIA does not.

In comparing ProPept-MT, we evaluated the multi-task model’s ability to predict retention time, ion intensity, and ion mobility for both unmodified and phosphorylated peptides. ProPept-MT was compared with ProPept-ST and DeepPhospho for phosphorylated peptides, and with ProPept-ST and DeepDIA for unmodified peptides. Unlike DeepPhospho and DeepDIA, which train and predict each task separately, ProPept-MT employs a multi-task approach. Due to the lack of ion mobility information in the datasets from comparative model studies, we used our curated datasets for this comparison. Overall, the use of different datasets and comparison modes ensures a fair and comprehensive assessment, meeting the requirements of each model.

Critical to peptide analysis are features such as fragment ion intensity, IM, and RT, which are effectively captured by our model through a nuanced training strategy that utilizes input data and noise from various tasks. This combined training approach helps mitigate the divergent noise inherent in different tasks, thereby improving learning outcomes and enhancing model robustness. In other words, by leveraging the inherent information contained within peptide sequences and precursor charge states, ProPept-MT was effectively trained on fragment ion intensity, RT, and IM within a multi-task learning framework. This advanced approach achieved a form of data augmentation, enabling each task to learn from a richer set of information rather than being confined to the specific data each task individually possessed, while also preventing overfitting. Additionally, ProPept-MT engages a hard parameter-sharing mechanism to embed the data representations of each task into a unified semantic space, followed by the application of a task-specific layer to extract task-specific representations for each task [52,53]. This approach significantly reduces memory consumption and eliminates redundant learning of information in shared layers, ultimately leading to higher inference speed and shorter training times.

Experimental results indicated that ProPept-MT outperformed single-task training, demonstrating robust enhancements in prediction accuracy. This suggested a certain degree of task interrelatedness among the three predicted peptide attributes, allowing each task to update parameters in a similar direction. By simultaneously optimizing multiple related tasks, ProPept-MT leveraged the interdependencies among them, ensuring that the intrinsic correlations within the data were effectively preserved and utilized. Furthermore, the fine-tuning process showcased the model’s flexibility, enabling ProPept-MT to seamlessly adapt to various types of LC and gradient lengths. This adaptability ensured that ProPept-MT could be applied to different experimental setups, enhancing its utility and performance across diverse proteomics research scenarios.

ProPept-MT manifested extensive potential applications. It accurately predicted the ion intensity, RT, and IM of peptides, facilitating the construction of 4D DIA spectral libraries. This capability aided in protein identification and quantitative analysis, enhancing the reliability of data and experimental efficiency. Moreover, we believe that ProPept-MT, through its precise analysis of proteomic mass spectrometry data, can uncover additional peptides and proteins, thereby providing valuable tools for fundamental scientific research, drug development, and disease treatment. We will continue to expand upon this research in the future.

Despite ProPept-MT’s improved prediction performance, negative transfer occurs during training. Analysis of the training loss curve reveals a step-like distribution of the losses for each task, with fragment ion intensity showing the smallest loss and IM the largest. This bias causes the model to prioritize reducing the loss of fragment ion intensity over RT and IM, potentially leading to sustained outstanding performance with regard to fragment ion intensity but the gradual deterioration of RT and IM performance. Furthermore, evaluating the performance improvement of each training epoch reveals challenges in selecting an optimal set of model parameters that perform best on each task. This issue underscores the need for further refinement. As a result, ongoing research focuses on alternative deep learning approaches to address these challenges and improve the prediction of additional peptide features. Future work also involves integrating spatial protein structures into training data to predict specific modification sites.

## Figures and Tables

**Table 1:** Performance of ProPept-ST under different parameters. “d_model” is the dimension of the embedded vector. “n_head” is the number of attention heads calculated in parallel in the multi-head self-attention module. “d_ff_lstm” is a hidden layer dimension in the lstm layer. “n_lstm” is the number of layers of lstm.

d_model	n_head	d_ff_lstm	n_lstm	MedAE
256	8	512	1	0.715
256	8	512	2	0.732
500	10	512	1	0.723
500	10	512	2	0.720
256	8	256	1	0.724
256	8	256	2	0.768
500	10	256	1	0.722
500	10	256	2	0.755

**Table 2:** Performance metrics of ProPept-MT on benchmark datasets.

Data Name	Metrics/Model	Retention Time					Ion Intensity			Ion Mobility		
		$R^{2}$	$\Delta t_{95\%}$	MedAE	IQR	PCC	SA	PCC	DP	$R^{2}$	PCC	$\Delta t_{95\%}$
H1_DDA	DeepPhospho	0.975	6.588	1.157	2.315	0.990	0.852	0.958	0.973	-	-	-
	ProPept-ST	0.9805	5.200	0.643	1.323	0.990	0.870	0.9657	0.979	0.977	0.9887	**0.0961**
	**ProPept-MT**	**0.9810**	**4.974**	**0.598**	**1.264**	**0.991**	**0.872**	**0.9663**	**0.980**	**0.978**	**0.9892**	0.0963
H2_DIA	DeepPhospho	0.989	5.481	1.019	2.046	0.997	0.805	0.889	0.953	-	-	-
	ProPept-ST	**0.998**	**2.333**	**0.319**	**0.638**	**0.999**	0.817	0.900	0.959	**0.986**	**0.9931**	**0.062**
	**ProPept-MT**	0.997	2.807	0.395	0.772	0.9986	**0.820**	**0.901**	**0.960**	0.984	0.9928	0.063
H3_DIAP	DeepPhospho	0.986	11.428	2.103	4.111	0.997	0.786	0.872	0.944	-	-	-
	ProPept-ST	**0.9952**	7.045	**0.753**	**1.508**	**0.99764**	0.797	0.882	0.9497	**0.985**	**0.9924**	**0.0614**
	**ProPept-MT**	0.9951	**6.973**	0.870	1.713	0.99763	**0.798**	**0.889**	**0.9502**	0.984	0.9922	0.0617
H4_DDAp	DeepPhospho	0.976	10.524	1.915	3.678	0.990	0.809	0.928	0.955	-	-	-
	ProPept-ST	0.9835	6.447	**0.715**	1.424	0.9918	0.831	0.941	0.965	0.971	0.9856	0.102
	**ProPept-MT**	**0.9839**	**6.203**	0.730	**1.422**	**0.9919**	**0.835**	**0.945**	**0.967**	**0.972**	**0.9862**	**0.099**
H5_DDAp	DeepPhospho	0.980	12.658	2.408	4.822	0.993	0.819	0.935	0.960	-	-	-
	ProPept-ST	0.987	9.249	**0.945**	**1.886**	0.9935	**0.8324**	0.940	**0.966**	**0.961**	**0.981**	**0.100**
	**ProPept-MT**	**0.988**	**8.699**	1.077	2.142	**0.9939**	0.8317	**0.941**	0.965	0.959	0.980	0.102
H6_DDAp	DeepPhospho	0.980	12.883	2.600	4.231	0.993	0.804	0.928	0.953	-	-	-
	ProPept-ST	**0.991**	6.519	0.820	1.660	**0.996**	0.814	0.934	0.958	**0.963**	**0.9815**	0.117
	**ProPept-MT**	0.990	**6.187**	**0.802**	**1.588**	0.995	**0.824**	**0.940**	**0.962**	0.960	0.9809	**0.113**
H7_DDAp	DeepPhospho	0.958	5.352	0.831	1.598	0.983	0.807	0.932	0.954	-	-	-
	ProPept-ST	0.977	2.853	0.318	0.650	0.988	0.823	0.941	0.961	0.982	0.991	0.080
	**ProPept-MT**	**0.980**	**2.255**	**0.294**	**0.587**	**0.990**	**0.838**	**0.950**	**0.968**	**0.986**	**0.994**	**0.067**
M1_DDAp	DeepPhospho	0.976	11.716	1.809	3.535	0.991	0.815	0.938	0.958	-	-	-
	ProPept-ST	**0.991**	6.519	0.820	1.660	**0.996**	0.814	0.934	0.958	0.963	0.981	0.117
	**ProPept-MT**	0.989	**5.498**	**0.702**	**1.423**	0.995	**0.834**	**0.949**	**0.966**	**0.982**	**0.992**	**0.077**
M2_DDAp	DeepPhospho	0.966	4.944	0.812	1.518	0.986	0.792	0.918	0.947	-	-	-
	ProPept-ST	0.980	3.050	0.367	0.755	0.990	0.807	0.927	0.955	0.941	0.973	0.112
	**ProPept-MT**	**0.982**	**1.668**	**0.243**	**0.483**	**0.991**	**0.827**	**0.941**	**0.963**	**0.955**	**0.978**	**0.090**

**Table 3:** Comparison of performance metrics between ProPept-MT and DeepDIA.

Data Name	Metrics/Model	Retention Time					Ion Intensity(2+)			Ion Intensity(3+)			Ion Mobility		
		$R^{2}$	$\Delta t_{95\%}$	MedAE	IQR	PCC	SA	PCC	DP	SA	PCC	DP	$R^{2}$	PCC	$\Delta t_{95\%}$
H1_DDA	DeepDIA	0.975	7.132	0.974	1.962	0.987	0.802	0.950	0.952	0.721	0.900	0.905	-	-	-
	ProPept-ST	0.980	5.422	0.670	1.399	0.9902	0.847	0.959	0.971	0.791	0.925	0.947	0.974	0.987	0.106
	**ProPept-MT**	**0.981**	**4.823**	**0.662**	**1.340**	**0.9905**	**0.866**	**0.968**	**0.978**	**0.817**	**0.943**	**0.959**	**0.976**	**0.988**	**0.100**
H2_DIA	DeepDIA	0.994	4.214	0.590	1.178	0.997	0.738	0.913	0.917	0.708	0.893	0.897	-	-	-
	ProPept-ST	**0.9973**	**2.817**	**0.381**	**0.770**	**0.999**	0.812	0.894	0.957	0.792	0.865	0.947	**0.984**	**0.9921**	**0.066**
	**ProPept-MT**	0.9970	2.993	0.426	0.852	0.998	**0.821**	**0.907**	**0.961**	**0.794**	**0.875**	**0.948**	0.983	0.9918	0.069
H3_DIAp	DeepDIA	0.979	15.410	1.964	3.909	0.990	0.692	0.880	0.885	0.673	0.867	0.871	-	-	-
	ProPept-ST	0.992	9.413	1.134	2.282	0.996	**0.788**	0.8507	**0.945**	0.7660	0.837	0.9332	0.979	0.990	0.073
	**ProPept-MT**	**0.993**	**8.301**	**0.939**	**1.873**	**0.997**	0.783	**0.8513**	0.942	**0.7664**	**0.852**	**0.9335**	**0.982**	**0.991**	**0.068**
H4_DDAp	DeepDIA	0.973	14.720	1.930	3.910	0.986	0.746	0.916	0.921	0.668	0.861	0.867	-	-	-
	ProPept-ST	0.984	8.243	0.992	1.972	0.992	0.805	0.928	0.954	0.750	0.891	0.924	0.960	0.980	0.126
	**ProPept-MT**	**0.986**	**6.685**	**0.865**	**1.729**	**0.993**	**0.819**	**0.938**	**0.960**	**0.774**	**0.913**	**0.937**	**0.967**	**0.984**	**0.l13**
H5_DDAp	DeepDIA	0.983	12.850	2.372	3.419	0.992	0.771	0.933	0.936	0.692	0.880	0.885	-	-	-
	ProPept-ST	0.989	8.484	0.942	1.888	0.9948	0.819	0.940	0.960	0.765	0.897	0.933	0.960	0.981	0.104
	**ProPept-MT**	**0.991**	**7.225**	**0.850**	**1.705**	**0.9954**	**0.839**	**0.952**	**0.968**	**0.798**	**0.928**	**0.950**	**0.969**	**0.984**	**0.086**
H6_DDAp	DeepDIA	0.918	21.389	2.456	4.928	0.958	0.670	0.861	0.869	0.657	0.853	0.859	-	-	-
	ProPept-ST	0.942	16.991	1.924	3.857	0.971	0.754	0.873	0.926	0.765	0.905	0.933	0.923	0.963	0.159
	**ProPept-MT**	**0.973**	**6.491**	**0.705**	**1.397**	**0.987**	**0.780**	**0.928**	**0.947**	**0.791**	**0.928**	**0.947**	**0.940**	**0.971**	**0.145**

## Data Availability

Open source after receiving articles such as source programs and data.

## Supplementary Materials

The following supporting information can be downloaded at: https://www.mdpi.com/article/10.3390/ijms25137237/s1.
