# Supplementary material for: ProPept-MT: A Multi-Task Learning Model for Peptide Feature Prediction
Source: Int J Mol Sci. 2024 Jun 30;25(13):7237. doi: 10.3390/ijms25137237 (PMC11241495; doi:10.3390/ijms25137237)
Supplement: Supplementary file 1 [file ijms-25-07237-s001.zip › ijms-3054902-supplementary.pdf]

Supplementary Material

Guoqiang He [1,2,†], Qingzu He [3,†], Jinyan Cheng [2], Rongwen Yu [2], Jianwei Shuai [2,*] and Yi Cao [2,*]

[1] Postgraduate Training Base Alliance, Wenzhou Medical University, Wenzhou 325000, China; wuhewuhe1999@wmu.edu.cn
[2] Wenzhou Institute, University of Chinese Academy of Sciences, Wenzhou 325000, China; chengjy@wiucas.ac.cn (J.C.); rwyu@ucas.ac.cn (R.Y.)
[3] Department of Physics, and Fujian Provincial Key Laboratory for Soft Functional Materials Research,
Xiamen University, Xiamen 361005, China; qingzuhe@stu.xmu.edu.cn
* Correspondence: shuaijw@wiucas.ac.cn (J.S.); caoyi@nju.edu.cn (Y.C.)
† These authors contributed equally to this work.

Figure of content

- Figure S1. Performance of ProPept-ST in predicting retention time.
- Figure S2. Compare the performance of ProPept-ST in predicting retention time with and without fine tuning.
- Figure S3. Performance of ProPept-MT in predicting retention time on benchmark datasets.
- Figure S4. Compare the performance of ProPept MT and DeepPhospho in predicting retention time and fragment ion intensity on benchmark datasets.
- Figure S5. Performance of ProPept-MT in predicting ion mobility on benchmark datasets.

Figure S1. Performance of ProPept-ST in predicting retention time.

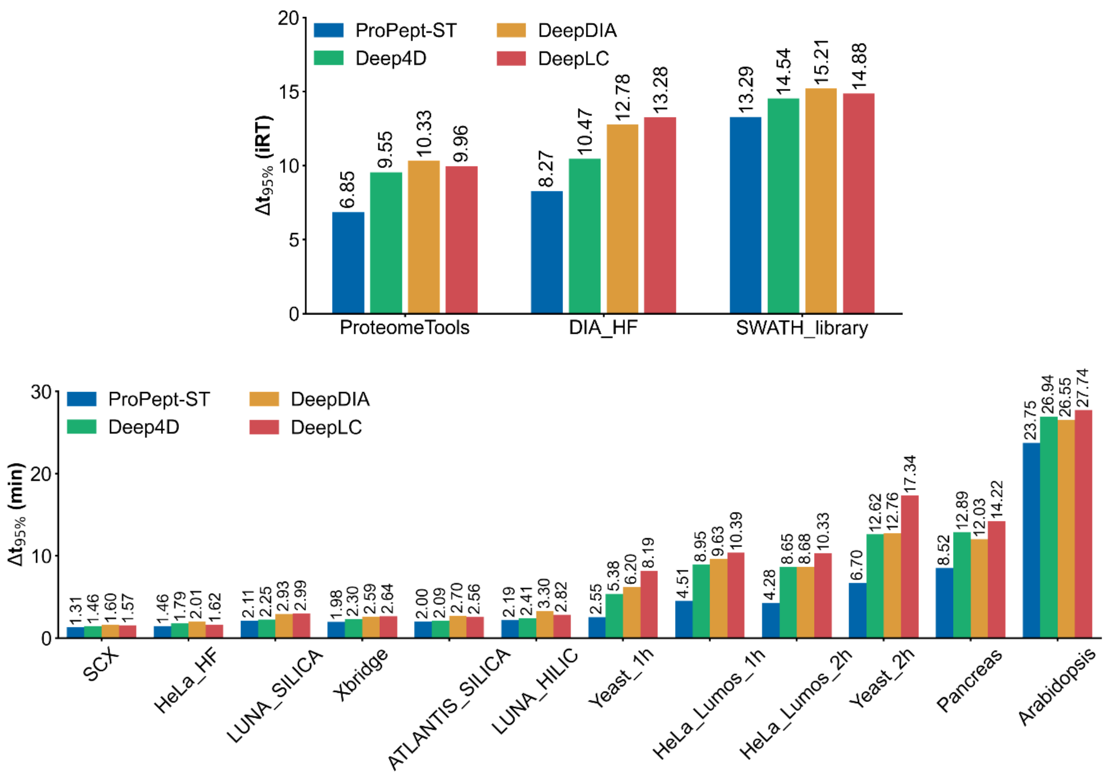

Figure S1. Performance of ProPept-ST in predicting retention time. Comparing the accuracy of peptide iRT (top panel) and RT (bottom panel) predictions among various models using the $\Delta t_{95\%}$ metric.

Figure S2. Compare the performance of ProPept-ST in predicting retention time with and without fine tuning.

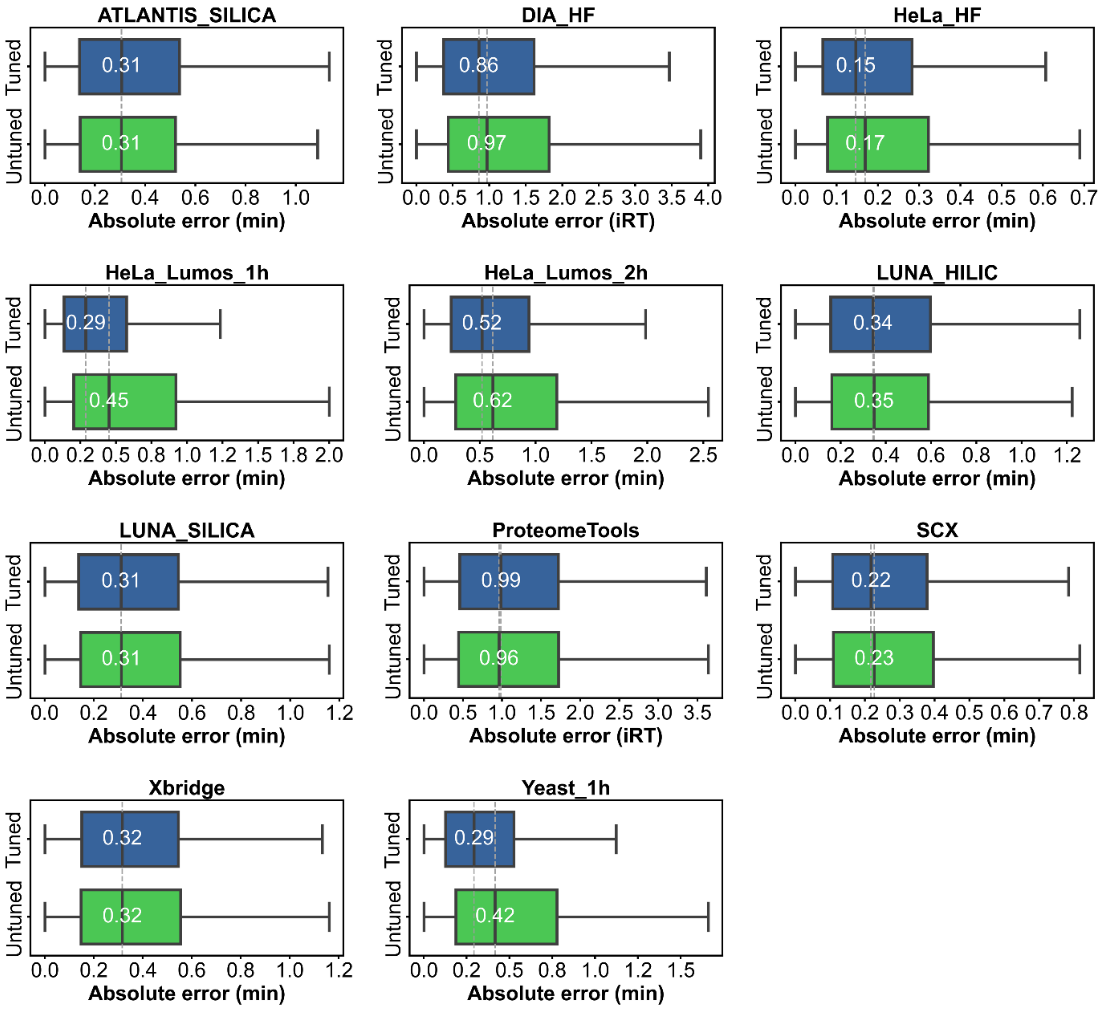

Figure S2. Compare the performance of ProPept-ST in predicting retention time with and without fine tuning. Among the 14 test datasets, the median absolute error in fine-tuned RT is 71.4% better than that of non-fine-tuned results, with only 0.07% exhibiting worse performance than the non-fine-tuned results, highlighting the significant benefits of fine-tuning in RT prediction.

Figure S3. Performance of ProPept-MT in predicting retention time on benchmark datasets.

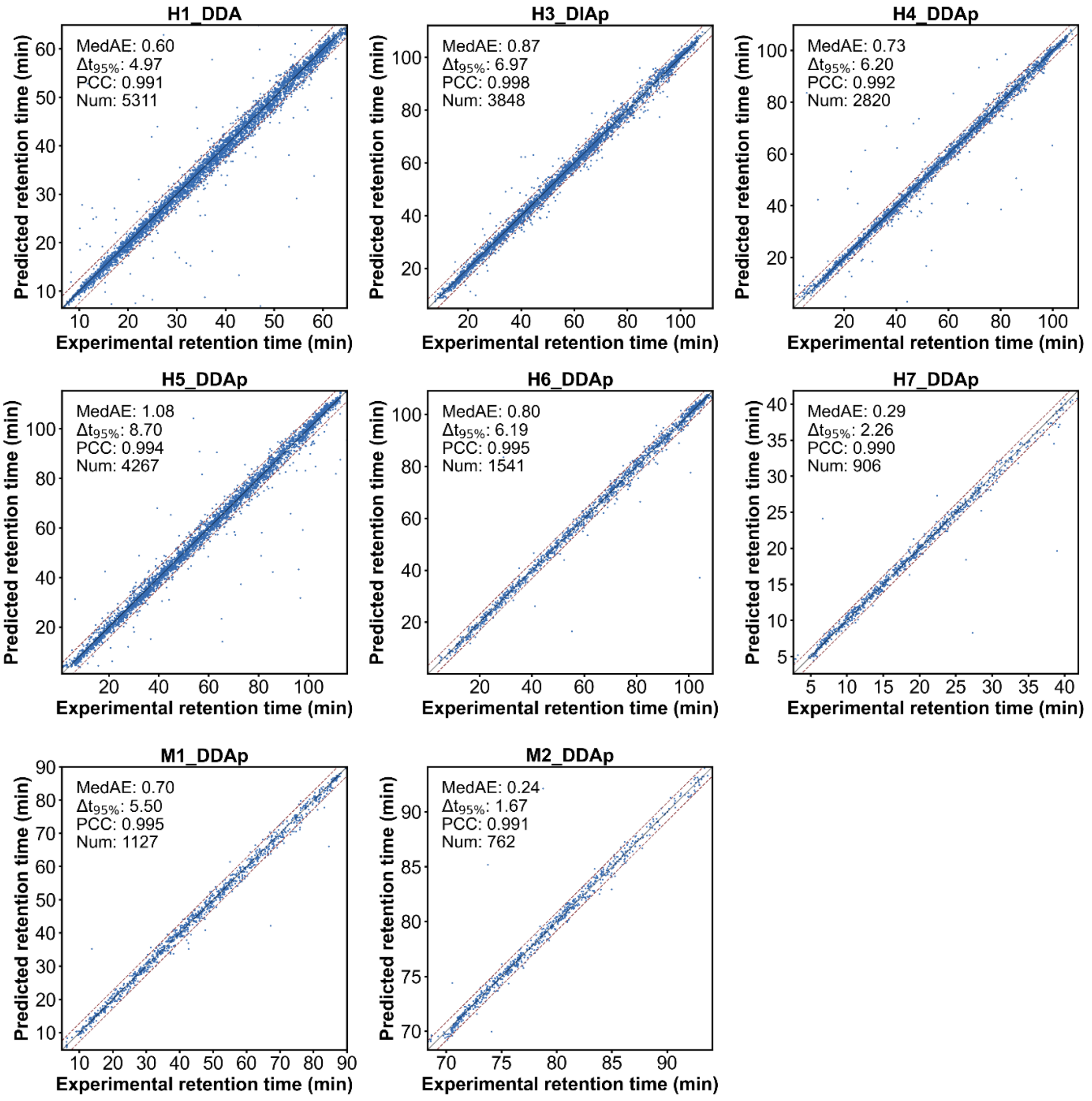

Figure S3. Performance of ProPept-MT in predicting retention time on benchmark datasets. The scatter plot illustrates the predicted RT values of ProPept-MT compared to the experimentally observed values on benchmark datasets, including metrics such as median absolute error (MedAE), $\Delta t_{95\%}$, PCC and the number of samples in the test set.

Figure S4. Compare the performance of ProPept MT and DeepPhospho in predicting retention time and fragment ion intensity on benchmark datasets.

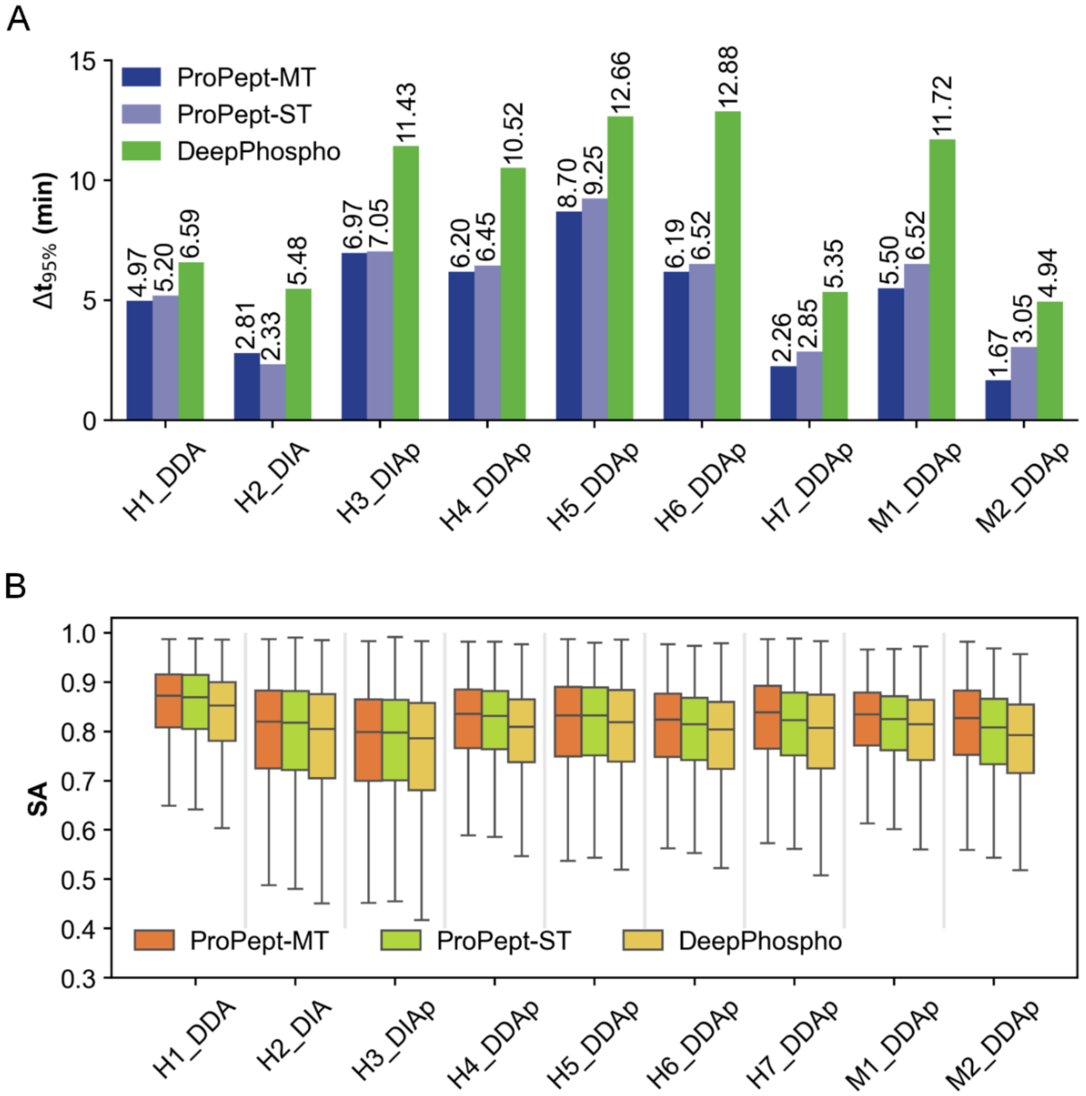

Figure S4. Performance of ProPept-MT in predicting ion mobility on benchmark datasets. (A) Comparing the accuracy of peptide RT predictions among various models based on different datasets. (B) Distribution of the SA index for the prediction of fragment ion intensity by various models on benchmark datasets.

Figure S5. Evaluating the IM prediction performance of ProPept-MT on benchmark datasets.

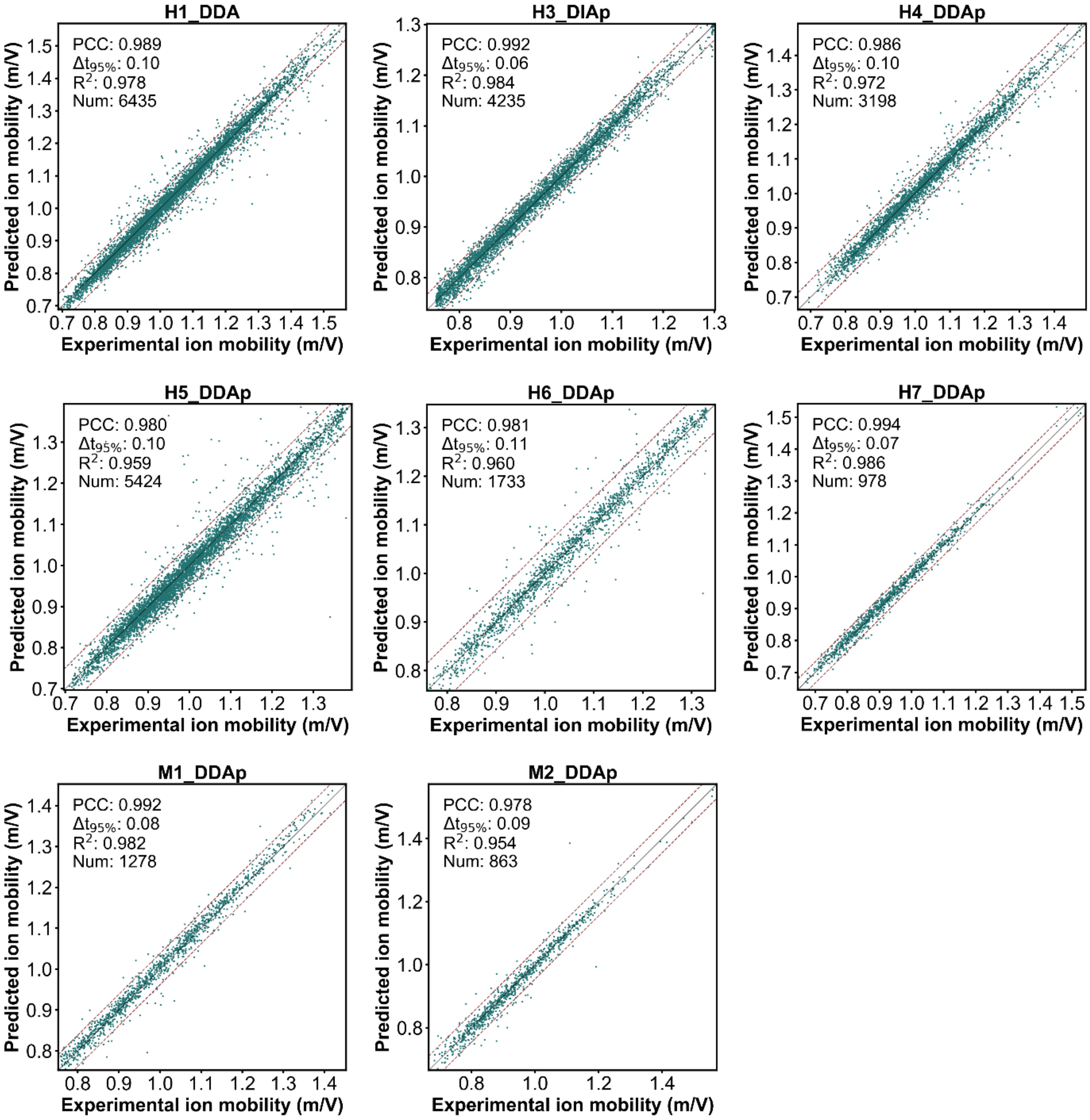

Figure S5. Performance of ProPept-MT in predicting ion mobility on benchmark datasets. The scatter plot illustrates the predicted IM values of ProPept-MT compared to the experimentally observed values on benchmark datasets, including metrics such as PCC, $\Delta t_{95\%}$, R^2 and the number of samples in the test set.
